# A Brief Patient-Reported Outcomes Quality of Life (PROQOL) Instrument to Improve Patient Care

**DOI:** 10.1371/journal.pmed.1001548

**Published:** 2013-11-12

**Authors:** Jennifer L. Ridgeway, Timothy J. Beebe, Christopher G. Chute, David T. Eton, Lacey A. Hart, Marlene H. Frost, Daniel Jensen, Victor M. Montori, John G. Smith, Steven A. Smith, Angelina D. Tan, Kathleen J. Yost, Jeanette Y. Ziegenfuss, Jeff A. Sloan

**Affiliations:** 1 Department of Health Sciences Research, Mayo Clinic, Rochester, Minnesota, United States of America; 2 Center for the Science of Health Care Delivery, Mayo Clinic, Rochester, Minnesota, United States of America; 3 Department of Medical Oncology, Mayo Clinic, Rochester, Minnesota, United States of America; 4 Public Health Services, Olmsted County Public Health, Rochester, Minnesota, United States of America; 5 Department of Endocrinology, Mayo Clinic, Rochester, Minnesota, United States of America; 6 Data Collection Center, HealthPartners Institute for Education and Research, Minneapolis, Minnesota, United States of America

## Abstract

Jeff Sloan and colleagues describe the development of the Patient-Reported Outcomes Quality of Life (PROQOL) instrument, which captures and stores patient-recorded outcomes in the medical record for patients with diabetes.

*Please see later in the article for the Editors' Summary*

**Summary Points**

- Patient-reported outcomes (PROs) provide a unique method of including the patient perspective at the point of clinical care. Many survey instruments have been developed to collect PROs, but some are lengthy and there are few examples of clinically integrated PRO collection to facilitate care management.
- The Patient-Reported Outcomes Quality of Life project (PROQOL) developed an instrument for patients with diabetes that systematically captures PROs at the health care visit, stores them in the medical record, and makes them available to the health care team, including members of public health departments.
- Patients are able to report concerns on less commonly discussed issues that may impact health management, such as social factors and personal relationships. The health care team develops a set of “suggested actions” to respond to a broad set of issues that impact patients' diabetes management within and outside the formal health care system.
- PROQOL was developed with input from patients and health care providers. This input was critical to the final design and resulted in practical lessons learned for organizations interested in integrating the patient's perspective into care management.
- Next steps include pilot testing in the 11-county region of southeast Minnesota and expansion to other patient populations and conditions.

## Introduction: The Challenge

Health care providers rely on a host of clinical metrics like lab values as they design care plans for their patients, but some information about the patient's health is best gathered through patient self-report. However, few studies to date have collected routine, real-time patient-reported outcomes (PROs) to inform care management, and those that do have used lengthy instruments or only reported results on a single occasion [1]–[3].

Recent quality- and performance-related standards, such as those put forth by the Patient-Centered Outcomes Research Institute (PCORI), the Institute for Healthcare Improvement, and the National Quality Forum, have given routine collection of PROs new emphasis in health care delivery research [4]. Collection of PRO data has the potential to enhance care management by helping providers understand not just whether a clinical value is within range but the impact of treatments on patients' lives. This can improve communication and patient engagement, and may ultimately result in higher patient satisfaction, increased adherence, and better outcomes [2],[5]–[7]. Collection of PROs may be especially important for patients with multiple chronic conditions, or for people whose treatment may be complex, involve multiple providers, and have a significant impact on daily life. The question addressed by this research is whether PRO assessments can be integrated into routine care in a meaningful way while being minimally burdensome to patients and providers [1],[2],[8],[9].

## The Proposed Solution

The Office of the National Coordinator for Health IT (www.healthit.gov) funded the Beacon Community Cooperative Agreement Program to support health information technology (HIT) development in 17 communities throughout the US. In November 2010, the South East Minnesota Beacon Community (SE MN Beacon) launched the Patient-Reported Outcomes Quality of Life (PROQOL) project to develop an instrument that electronically captures PROs from patients with diabetes at the point of care and electronically integrates them into the medical record. We aimed to build HIT across providers in the 11-county region, including public health, so that all members of a care team can understand the patient's health regardless of their physical location.

## PROQOL Development

Our team included expertise in psychometrics, survey design, quality of life (QOL) measurement, health literacy, and behavioral psychology. We met regularly with a SE MN Beacon advisory group made up of HIT professionals, endocrinologists, family physicians, and other health care providers. Our first step was identification of existing PRO survey instruments and item pools related to patient QOL [10]. We reviewed 12 instruments measuring diabetes-specific QOL, as well as nine instruments and item pools related to QOL in a broader patient population on the following quality criteria: potential for self-administration, psychometric properties (i.e., validity and reliability), numbers of literature citations, and length or average time to completion.

From this list, we retained eight diabetes-specific instruments for possible inclusion. Single items measuring health-related QOL but not specific to diabetes from the Linear Analogue Scale Assessments (LASA) and the Patient-Reported Outcomes Measurement Information System (PROMIS) short forms were also selected for further review. Together these instruments have more than 300 items in domains including well-being, psychological distress, physical function, fatigue, social relations, and other areas related to patient QOL.

While this process demonstrated the availability of existing measures, it left unanswered the question of how to prioritize items from this broad and lengthy list. To inform our next steps in item selection, we conducted discussion groups with providers and patients. Provider groups were held in three settings including two family practice sites (Winona Health in Winona, MN, and Olmsted Medical Center in Rochester, MN) and one public health visiting nurse program (Olmsted County Public Health, Rochester, MN). Participants included physicians, registered nurses, registered dieticians, certified diabetes educators, public health nurses, social workers, and clinic administrative staff. Patient input was gathered using discussion groups with members of a diabetes patient advisory group at Mayo Clinic (Rochester, MN) and a diabetes survivors group (Winnipeg, Canada). The following open-ended questions were asked in the provider groups, and similar questions were posed to the patient groups. Both groups were also asked about their experience with PRO collection and for input on optimal assessment length and frequency.

1. Consider the information you currently get directly from a person with diabetes. What information is most important to you when making decisions about the course of treatment or care?
2. What information would you like to get that you are currently not getting?
3. What information is most helpful to you in determining whether that person's health status has changed since the last time you saw them?
4. Imagine that you could observe the person with diabetes in their everyday life. What information would you hope to gain that would inform their course of treatment or care?

Participants discussed typical patient-reported information like blood sugar monitoring and diet, but they focused on how diabetes management is impacted by patients' social context, such as home life, finances, or emotional health. One provider group talked about workplace situations. Some patients were unable to test and eat on a recommended schedule because they could not leave the manufacturing line, impacting their ability to manage their diabetes. In another provider group, we heard that family and financial circumstances were negatively impacting diet options for their patient population.

Patients told us that these issues were important for diabetes care, but they did not always feel comfortable initiating a conversation as they worried it was inappropriate to discuss such things with their providers, who were more focused on symptoms and lab results. Several said a questionnaire could help bring new topics into their conversations with health care providers. For their part, providers talked about time-constrained visits. They wanted all of the information that would aid in care management, but getting it seemed overwhelming. They needed to get to the key points of the visit quickly and they often used general questions at the start of an appointment, like “How are things going?” to get at the patient's social context. Participants in both groups said five to ten questions were the preferred maximum.

After all three groups were completed, team members reviewed notes from the groups and then met to discuss and come to consensus on the most prevalent themes. The following domains emerged: personal relationships, monitoring health, emotional health, money, health behaviors, medicine, getting health care, work, and physical health. We scheduled a second round of discussion groups with providers to review these findings. Participants were presented with the list of the nine domains. We then presented a mock-up of a classic psychometric approach to gathering PROs. Psychometric theory suggests listing all possible aspects of a domain, and then constructing survey items to cover each aspect. Using the money domain as an example, we demonstrated how the respondent would be presented with money-related question items, such as “In the last 4 weeks, did you have trouble paying your medical bills?” or “Did you have trouble paying for your health insurance?” Branching patterns would lead the respondent to subsequent questions in order to eventually identify money-related issues that may be a concern for the patient. From there, the respondent would answer questions in the other domains.

Participants in our follow-up groups agreed that the domains we selected represented the most important patient-reported information, but they also felt that this traditional psychometric approach was not only impractical in terms of data collection burden, but it would also produce an amount of information that was unlikely to be consumable and actionable in a time-constrained clinical visit. Therefore we decided to focus on the domain that represented the patient's biggest concern at the time of the visit. The resulting question the PROQOL system puts to the patient is “Which of the following, if any, represents your single biggest concern right now?” which is similar to the “How are things going?” question many providers were using to start patient conversations.

**Figure 1:**
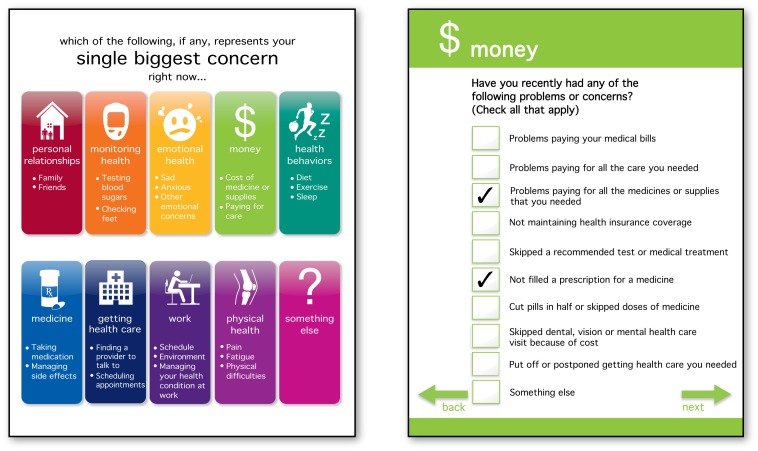
PROQOL domains and item checklist. Patients select their single biggest concern at that time from these domains (left). They are then presented with a checklist of items related to the selected domain, as in the money domain example presented here (right).

Participants also suggested changes to how the patient would identify issues within the selected domain. The classic psychometric approach, although valuable in the data it provides, can result in lengthy and time consuming instruments. For example, the Diabetes Care Profile (DCP) is divided into 16 profile scales with four to 19 questions per scale and takes approximately 30 to 40 minutes to complete [11]. Instead, we developed a checkbox approach whereby a patient, after selecting the domain that represents his or her single biggest concern at that time, is presented with a list of five to nine issues in that domain, selected from existing validated instruments (shown in Figure 1 using the money domain as an example). A “something else” category was retained to prompt patients to talk with providers about issues not covered by PROQOL.

**Figure 2:**
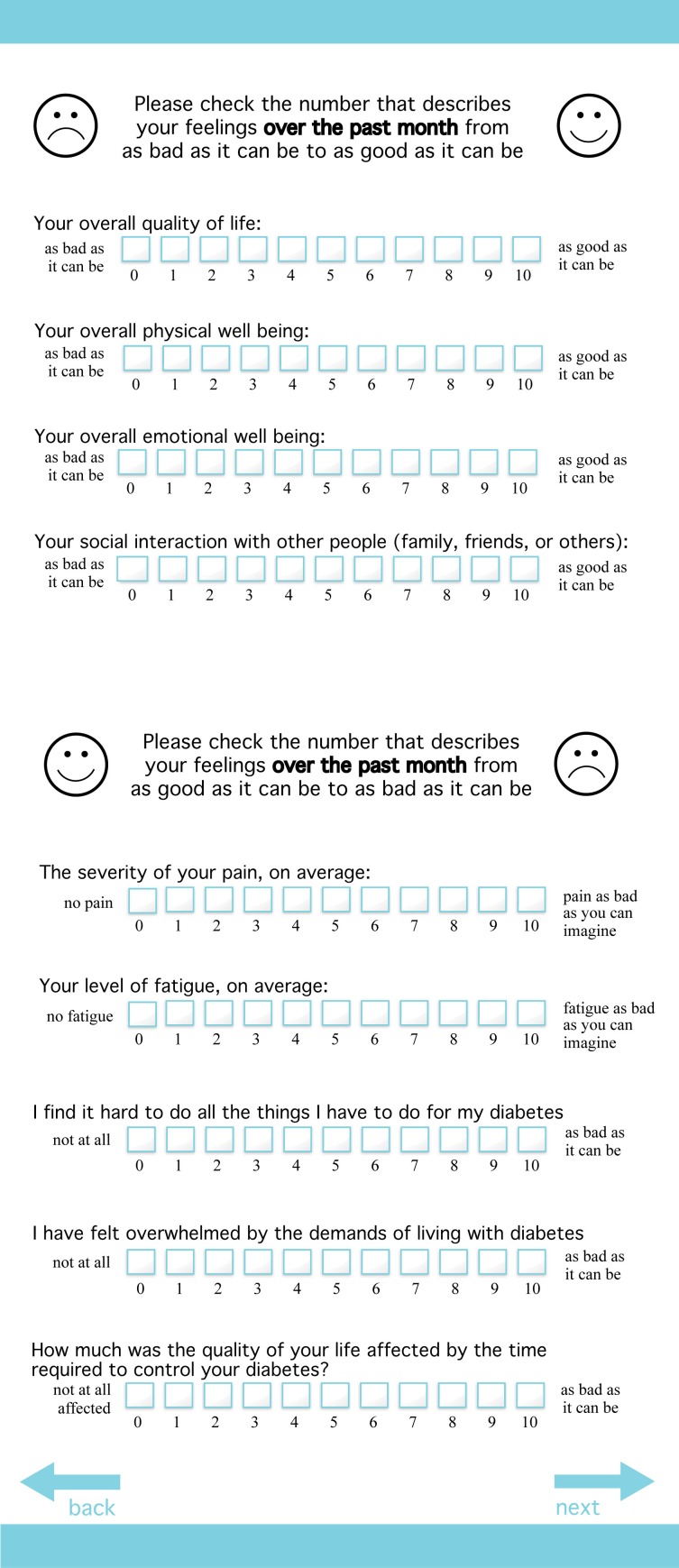
Core set of PROQOL questions. In addition to identifying their concerns at the time of survey completion, patients are also presented with this core set of quality of life-related questions.

**Figure 3:**
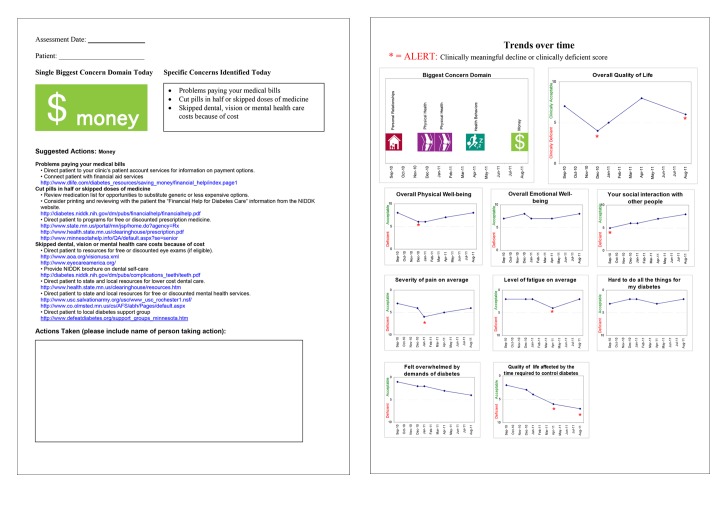
PROQOL report. PROQOL generates a report displaying the patient's single biggest concern (domain), the selected items in the domain, and suggested actions (left). The biggest concern and the results of the core set of questions are tracked over time. Graphs are used to display changes, and asterisks indicate meaningful change since the last report (a change of two or more points).

This approach allows providers to quickly focus on a single issue area, which may change from visit to visit depending on a patient's needs, but we also wanted a way to track standard QOL-related issues for the patient and the entire diabetes patient population over time. This led to development of a final set of scaled items presented to all patients, a process that was informed by a third round of provider discussion groups (Winona Health in Winona, MN, and Olmsted Medical Center in Plainview, MN) and an additional patient discussion group (Winona Health). As shown in Figure 2, the final items included six questions from the LASA, and one question each adapted from the DCP, the Diabetes D-39, and the Diabetes Distress Scale (DDS). The final report generated by the system displays the current concerns and responses to the final set of scaled items, as well as charts displaying changes over time, as shown in Figure 3.

## Clinical Realities

Organizations like the National Quality Forum suggest that assessing PROs like patient well-being can be key to evaluating quality of care for patients with multiple chronic conditions [12]. We were able to create a tool that included the most important PRO domains, as identified by providers and patients. It gets to the most important concern of a patient quickly and can open up the conversation to include less-frequently covered topics like social context. By putting results in the medical record, all members of the care team have access to it. From these perspectives, we have achieved the aims of collecting the most important PROs and coordinating care.

However, we still faced issues related to practical implementation. First, some providers continued to voice concern about the time it would require to discuss additional topics during the visit, even if they saw the value in it. They also noted concern about being presented with issues outside their realm of expertise. Our proposed solution was development of suggested actions to engage the entire clinical team, staff, and community resources. For each patient concern listed in the report, there is a related suggested action, an idea that grew out of the clinical pathways clinicians are accustomed to following. For example, a patient who indicates a problem paying for prescriptions might trigger a conversation with the provider about prescribing options, but the report would also provide links to any available financial resources, which could be discussed with another member of the team. Unique to PROQOL is that the suggested actions are a template that local health care teams can adapt to reflect local community resources and their specific team's structure or method of operation. Often these actions are referrals to a team member other than the clinician, such as a diabetes educator or a nutritionist, or a community resource. In some cases, the suggestion is simply to probe for greater detail on a concern, which gives the provider a better understanding of what is going on in a patient's life and how that may impact diabetes management. In this way we allow for discussion of important topics in the clinical visit without assuming that all subsequent actions must be taken by the clinician.

Second, developing a seamless integrated system that is easily shared by patients and providers is challenging. Local HIT and legal or organizational policies may vary between settings, complicating data sharing. Without the real-time exchange, PROQOL's power to foster collaboration that improves holistic patient care is compromised. The SE MN Beacon IT solutions to exchange electronic health information with clinical and public health entities across the region continue to evolve.

## Next Steps

The next step for this project is to continue addressing clinical realities, including HIT development, data sharing, and minimizing impact of the instrument on workflow. Pilot tests of PROQOL usability are currently being held in various settings in Minnesota and other Beacon Communities around the US. These pilot studies will be assessed qualitatively by interviewing providers and patients to determine issues related to workflow, burden, and perceived value of the information provided. We are also collecting information about which domains and items are selected most often and debriefing patients about their understanding of the items. Although PROQOL was developed for patients in the diabetes care setting, the underlying system and the vast majority of content is adaptable to other conditions. We are implementing PROQOL in other clinical areas for patients with complex care needs such as oncology and critical care.
